# Selection for Phase Variation of LOS Biosynthetic Genes Frequently Occurs in Progression of Non-Typeable *Haemophilus influenzae* Infection from the Nasopharynx to the Middle Ear of Human Patients

**DOI:** 10.1371/journal.pone.0090505

**Published:** 2014-02-28

**Authors:** Kate L. Fox, John M. Atack, Yogitha N. Srikhanta, Anja Eckert, Laura A. Novotny, Lauren O. Bakaletz, Michael P. Jennings

**Affiliations:** 1 Institute for Glycomics, Griffith University, Gold Coast, Queensland, Australia; 2 School of Molecular and Microbial Sciences, University of Queensland, St. Lucia, Brisbane, Queensland, Australia; 3 Centre for Microbial Pathogenesis, The Research Institute at Nationwide Children’s Hospital, Columbus, Ohio, United States of America; University of Padova, Medical School, Italy

## Abstract

Surface structures in *Haemophilus influenzae* are subject to rapid ON/OFF switching of expression, a process termed phase variation. We analyse tetranucleotide repeats controlling phase variation in lipo-oligosaccharide (LOS) genes of *H. influenzae* in paired isolates from both the nasopharynx and middle ears of paediatric patients with chronic or recurrent otitis media. A change in expression of at least one of the seven phase variable LOS biosynthesis genes was seen in 12 of the 21 strain pairs. Several strains showed switching of expression in multiple LOS genes, consistent with a key role for phase variable LOS biosynthetic genes in human infection.

## Introduction

Non-typeable *Haemophilus influenzae* (NTHi) strains cause otitis media (OM), sinusitis, conjunctivitis, and acute lower respiratory tract infections. OM is an infection of the middle ear resulting in middle ear effusion, fever, irritability, and inflammation of the tympanic membrane; it is the most common bacterial infection in infants and young children [1]. It is generally assumed that middle ear infection occurs when bacteria colonising the nasopharynx enter the middle ear space via ascension of the Eustachian tube [2].

Phase variation is the reversible, high frequency switching of gene expression. In *H. influenzae*, phase variation is commonly mediated by mutations in simple tandem DNA repeats that occur in genes, mainly encoding surface expressed virulence determinants [3]. Switching of expression of these genes allows the generation of a diverse population of phenotypically distinct bacteria. As organisms move from one microenvironment to another, such as during OM where organisms progress from the nasopharynx to the middle ear, a unique mixture of variants may be selected which are better adapted to survival [4].

**Figure 1:**
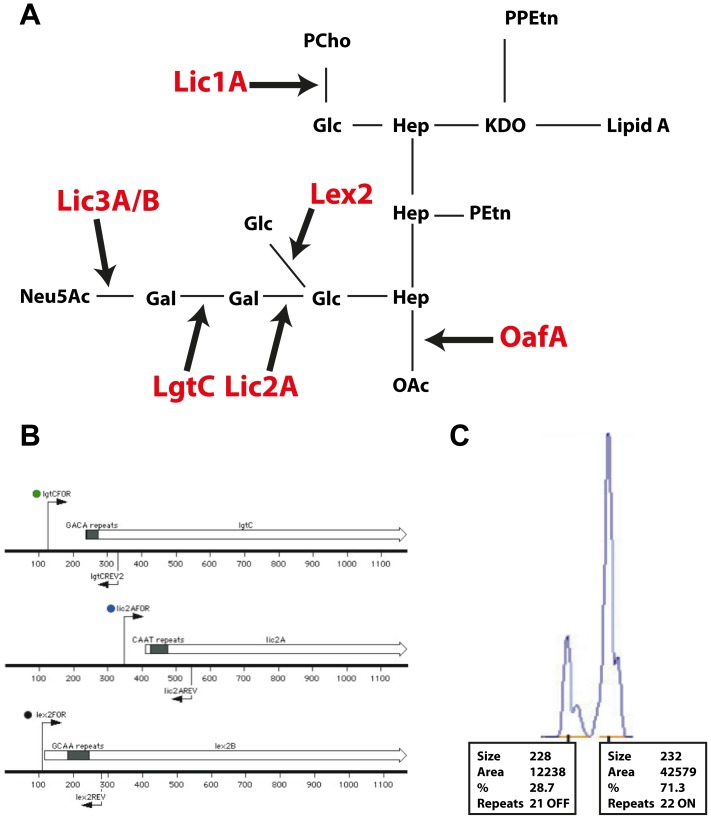
LOS biosynthesis pathway in *Haemophilus influenzae*, and examples of multiplex PCR and fragment analysis traces. (**A**) an illustration of the roles of the proteins encoded by the seven phasevariable genes studied in this work: Lic1 = phosphocholine transferase; Lic2A = galactosyltransferase; Lic3A and Lic3B = analogous sialyltransferases; Lex2 = glucosyltransferase; OafA = O-acetylase; LgtC = galactosyltransferase. The LOS contains 2-keto-3-deoxyoctulosonic acid (KDO); phosphoethanolamine (PEtn); pyrophosphoethanolamine (PPEtn); heptose (Hep); galactose (Gal); glucose (Glc); phosphocholine (PCho); and O-acetyl group (OAc); (**B**) an example of a multiplex PCR reaction utilized in this study using three gene specific primer pairs, with each pair containing a forward primer with a specific fluorescent label: FAM (6-carboxyflourescein), VIC, or NED; and (**C**) an example of a typical fragment analysis trace generated by a fluorescent PCR reaction separated using the Genescan system.

In *H. influenzae* many of the known phase variable genes encode proteins involved in lipo-oligosaccharide (LOS) biosynthesis (Figure 1A). These include four genes containing a 5′CAAT intergenic repeat tract: *lic1*, encoding a phosphocholine transferase [5], *lic2A*, encoding a galactosyltransferase [6] and *lic3A* and *lic3B*, encoding related sialyltransferases [3], [7]; two genes containing a 5′GCAA intergenic repeat tract: *lex2*, encoding a glucosyltransferase [8], and *oafA*, encoding an O-acetylase [9]; and one gene containing a 5′GACA intergenic repeat tract: *lgtC*, encoding a galactosyltransferase [10] (Figure 1A). Recent studies have observed selection for NTHi LOS phase variation after exposure to human serum [11], and in the colonisation of the human nasopharynx in a new human model of NTHi colonisation [12]. Here we report an analysis of the phase variation of seven LOS biosynthetic genes in a collection of paired clinical isolates from the nasopharynx and middle ear of paediatric patients with chronic or recurrent OM, in order to look for evidence of selection for LOS phase variants that might have emerged during the transition from nasopharyngeal colonisation to overt infection of the middle ear.

## Materials and Methods

### Bacterial Isolates and Growth Conditions

Thirty sets of paired NTHi isolates (n = 65 individual isolates) were used in this study, as described in Table S1. These isolates represent two strain collections recovered from paediatric patients undergoing tympanostomy tube insertion for chronic and/or recurrent OM at Nationwide Children’s Hospital, Columbus, Ohio. Sixteen clinical pairs (n = 37 strains) were recovered during the period 1982–1986, and fourteen (n = 28 strains) were recovered during the period 2004–2008. All isolates were recovered from patients undergoing surgery for tympanostomy tube insertion after obtaining written informed consent from patient guardians. Consent forms and study protocols were approved by the Nationwide Children’s Hospital Institutional Review Board.

Prior to entry into either strain collection, all strains were confirmed to be NTHi and were maintained in liquid nitrogen after minimal passage on artificial media. In order to conduct fragment size analysis of repeat regions, NTHi isolates were grown at 37°C on Brain Heart Infusion (BHI) plates, prepared with 1% (v/v) agar and supplemented with 10% (v/v) Levinthal base.

### DNA Preparation, Manipulation and Analysis

Bacterial genomic DNA was isolated and purified using the GenElute kit (Sigma-Aldrich), and PCR products were purified using a PCR purification kit (Invitrogen). PCR primers were purchased from Sigma Proligo and Applied Biosystems. The primers described in Table S2 were used for PCR and sequencing of the repeat region of genes, and labelled versions of the forward primers were used for multiplex PCR and DNA fragment analysis. Primers P6-F and P6-R [13] were used to amplify the *ompP6* gene, whereas primers him6A and him11 [14] were used to amplify the variable region of the *mod* gene (Table S3). PCR of the *ompP2* and *ompP5* genes was carried out using primers pairs P2-F & P2-R, and P5-F & P5-R, respectively, designed to amplify all the coding regions of the OMP P2 and OMP P5 genes, and based on those used previously to characterise these genes in *H. influenzae* [15], [16] (Table S3). Sequencing reactions were prepared using PCR products as template and Big-Dye sequencing kit (Perkin Elmer). Samples were analysed using a 3130xl Capillary Electrophoresis Genetic Analyser (Applied Biosystems International). Data were analysed using MacVector (version 9.0). To analyse DNA fragment sizes, multiplex PCR products amplified using primer sets in which the forward primers were labelled with either 6-carboxyfluorescein (6-FAM), NED or VIC, (an example is presented in Figure 1B), were analysed using the GeneScan system (Applied Biosystems International; an example is presented in Figure 1C).

## Results and Discussion

Work by Murphy and colleagues [13] showed that isolates of apparent *H. influenzae* sampled from patients with chronic obstructive pulmonary disease (COPD) were in fact *Haemophilus haemolyticus*. They also reported that 27–40% of isolates of apparent *H. influenzae* recovered from sputum samples or nasopharyngeal swab specimens were *H. haemolyticus*. To confirm that the isolates used in this study were *H. influenzae*, the gene encoding P6 was PCR amplified and sequenced [13] from all of the clinical isolates. Analysis of the DNA sequence, in combination with biochemical tests and lack of reactivity with immune serum directed at *H. influenzae* capsule types a–f (data not shown), confirmed that all isolates used in this study were indeed NTHi. These findings suggest that unlike in patients with COPD, *H. haemolyticus* does not colonise the nasopharynx of paediatric patients with OM as frequently. Furthermore, to allow us to exclude pairs of isolates that were not the same strain of *H. influenzae*, the variable region of the methyltransferase (*mod*) gene [14] was sequenced and compared. Of the 30 sets of proposed paired isolates (Table S1), seven did not share the same *mod* variable region sequence. These sets of isolates were thus excluded from the study. Strain pairs were also examined for close correspondence of tetranucleotide repeat numbers for each of the LOS biosynthetic genes. As a result of this analysis, one additional set of isolates (74N/E) was excluded from the study due to the fact that all repeat regions studied had vastly different repeat tract lengths, indicating it is not likely to be a true strain pair (Figure S1). Of the remaining 22 pairs of isolates that showed the same *modA* allele, and contained similar numbers of repeats in all LOS biosynthetic genes, as well as similar numbers of repeats in the *modA* and *hsdM* genes (Table S1, Figure S1), we sequenced the genes encoding the major outer-membrane proteins (OMPs) P2 and P5. These genes have been well characterised and contain regions of high homology, as well as highly variable regions and are established methods for typing NTHi strains [15], [16]. Of these 22 pairs of isolates, 21 contained identical OMP P2 and OMP P5 genes. Thus we can confidently say these 21 pairs of isolates are matched strains (Table S1; Figure S1).

**Figure 2:**
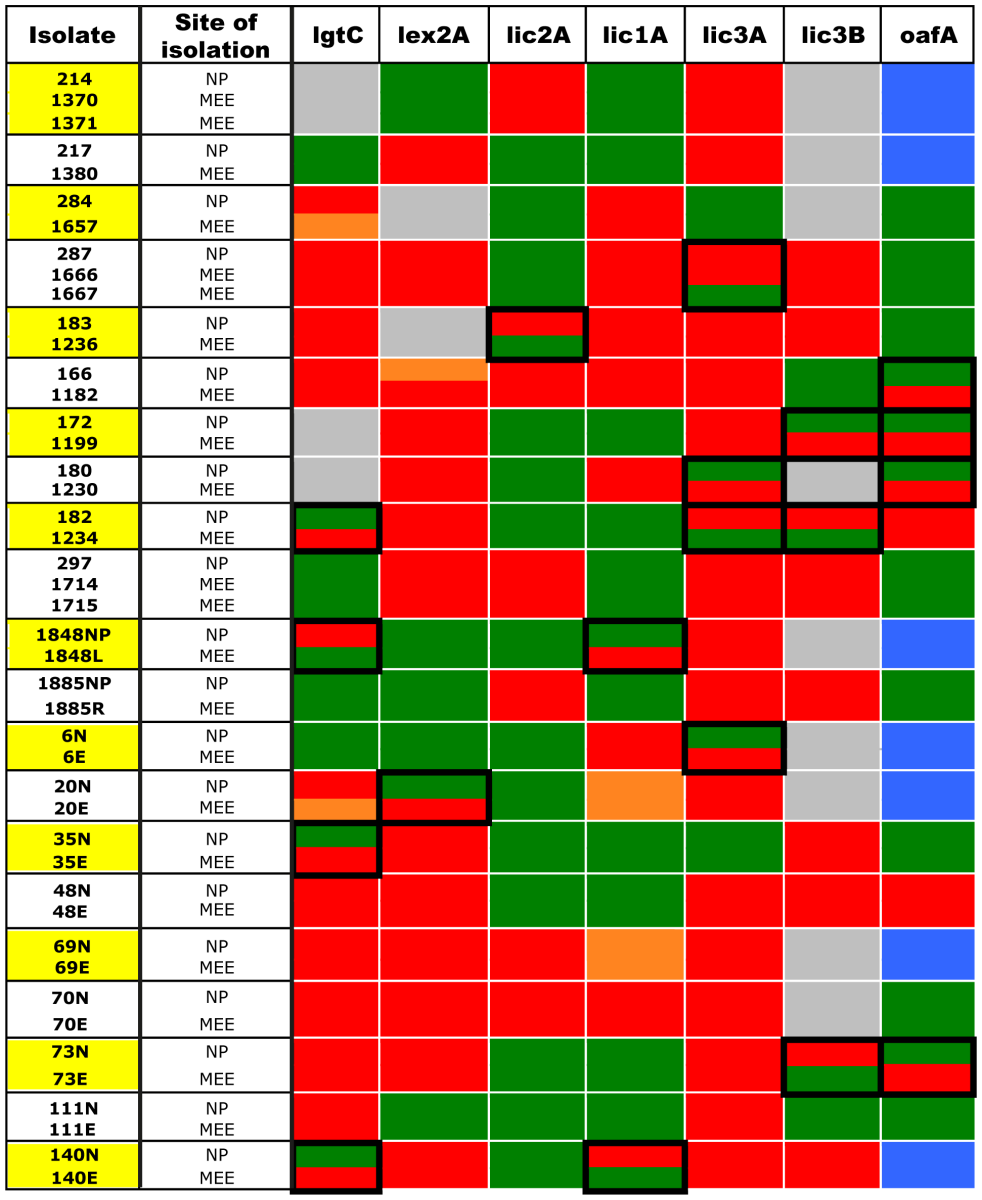
Heat map showing the ON/OFF status of the phase variable LOS biosynthesis genes. Green = >70% ON; Red = >70% OFF; Orange = mixed ON/OFF. Switching events are highlighted with a black box. Grey = no gene present; blue = gene is not phasevariable.

Fragment analysis was performed on the PCR products amplified from the remaining 21 paired strain sets. The distribution of the peak sizes obtained revealed the proportion of the population that is ON or OFF for a particular LOS biosynthesis gene. The results shown in Figure 2 indicate whether genes were ON (>70% ON; green), OFF (>70% OFF; red) or mixed ON and OFF (orange). This was determined from the number of tetranucleotide repeats (based on amplicon peak size; for example see Figure 1C), and on previous studies that have demonstrated the relationship between tetranucleotide repeat number and gene expression [17].

Using these 21 confirmed sets of paired isolates of NTHi, with each strain containing a maximum of seven phase variable LOS biosynthetic genes, a total of 147 potential phase variation events could occur. However, in some cases no PCR product was observed, indicating certain genes were not present in certain strains. For example, *lic3B* was not found in nine of the 21 paired isolates, and *lgtC* was absent from three of the 21 paired isolates. This result was anticipated, as many of the genes involved in LOS biosynthesis in NTHi are not found in every strain. In other cases, the strain contained an allele of the LOS biosynthesis gene that had no simple tandem repeats. This outcome was also anticipated, as *oafA,* for example, has previously been reported to occur as a non-phase variable allele in some NTHi strains [9]. Thus, excluding the absences of LOS genes in some strain pairs (14) and non-phase variable alleles (7; all *oafA*), a total of 126 opportunities remained wherein a potential phase variation event might be observed in the collection. In 19 of these 126 cases (15.1%) we obtained evidence that a phase variation event had occurred during passage from the nasopharynx to the middle ear.

Each gene that has evolved a phase variation mechanism must have a pro- and counter-selective pressure. Some of these selective pressures have been well characterised in *H. influenzae*. For example, the ChoP modification of LOS can serve as a target for C-reactive protein (CRP)-mediated killing of *H. influenzae* by the immune system [18], however in modification of LOS, ChoP acts as an adhesin for the Platelet Activating Factor receptor, important for colonisation [19]. In strong support of this observation, the recent study of Poole *et al* (2013) showed a consistent switch in Lic1 phosphocholine LOS transferase from OFF (in the inoculum) to ON during nasopharyngeal colonisation of human subjects, consistent with the proposed role in adherence to the airway epithelium [12].

Previous studies using a model system for analysis of *in vitro* [11], [20] and *in vivo* studies [12], [18], [21], [22], have used a common single strain inoculum, or only examined a single LOS gene in multiple NTHi strains [9], . The current study examined phase variation of multiple, phase variable LOS genes in independent paired clinical isolates from independent subjects. Furthermore, rather than observing phase variation in LOS biosynthetic genes over a specific time course from a defined inoculum, this study of clinical isolates did not have a common time line from colonisation to development of OM. Therefore, the history and stage of the infection and consequent host factors (immune response) likely to dictate selection and counter-selection for various LOS phase variation events are unknown variables in this study. The question addressed here is limited to providing evidence for distinct selection for LOS variation as an adaptation to two distinct host microenvironments at a single time point in the infection when the subjects presented for surgical management of chronic or recurrent OM. Despite these limitations, we observed evidence for selection of LOS phase variation in adaptation to these two microenvironments: a change in expression of at least one of the seven phase variable LOS biosynthesis genes surveyed was observed in 12 of the 21 strain pairs surveyed (57%; Figure 2); phase variation of two or more genes was observed in six of the 21 strain pairs surveyed (29%), and in the case of the OafA acetylase, we observed evidence for consistent selection in multiple subjects (Figure 2).

Our analysis reveals that *lic3A* OFF is the preferred state *in vivo*, regardless of the site of isolation (15 out of 21 strain pairs). However, as we did not observe a consistency of switching, the significance of this is beyond the particular scope of this paper. However, as sialyation of LOS has been shown to be important for serum resistance, persistence and biofilm formation by NTHi [24]–[26], this finding does merit further investigation. Of particular note is the observation that sialylated glycoforms of LOS appear to be particularly important during acute OM infection, when there is significant need to evade a strong host immune response. These glycoforms appear to be less important during chronic infections, which are characterised by a decreased immunological response [27], [28]. Thus, a preference for *lic3A* to be OFF in our samples may be a result of the stage of infection that these strains were isolated from, rather than an event resulting from a specific selective pressure.

In analysis of the *oafA* gene, we observed that seven of the 21 strain pairs (33%) contain an *oafA* gene with no repeats, and therefore are not phase variably expressed (blue boxes; Figure 2). Of the 14 that have repeats, eight were ON in the nasopharynx and remained ON in the middle ear. Two strain pairs were OFF in the nasopharynx and remained OFF in the middle ear, although one of these strain pairs (182/1234) had only three tetranucleotide repeats and is thus unlikely to switch at high frequency (Figure S1). Four strain pairs that were ON in the nasopharynx switched to OFF in the middle ear. No strains were observed that switched from OFF in the nasopharynx to ON in the middle ear (Figure 2). These data are consistent with a selective advantage for those individuals that have switched expression of *oafA* OFF upon transfer from the nasopharynx to the middle ear. Acetylation of LOS in several Gram-negative species has been associated with immunogenicity, with the acetyl group being a key component of epitopes responded to by the host [29], [30]; thus, phase variation of *oafA* to OFF, as seen with our data (Figure 2), may aid in evasion of this primed response in the middle ear. Moreover, and converse to this latter observation, the presence of acetylated LOS in NTHi has been associated with general serum resistance [9]. Thus, a change in host micro-environments upon transfer from the nasopharynx to the middle ear appears to select for a switching of expression of *oafA* from ON to OFF. Critical to this observation is that the reverse event (OFF in nasopharynx to ON in middle ear) was not observed. The severity of the clinical symptoms of the strains that switch *oafA* ON to OFF and their ‘success’ at colonising the middle ear when compared to those strains that remain ON, would confirm if the switch to OFF does indeed give a selective advantage in the middle ear.

## Supporting Information

Figure S1 **All data generated by fragment analysis of the seven phasevariable LOS synthesis genes, summarized in** **Figure 2** **.** In addition to the raw data presented as the heat map in Figure 2, the status of the P6 protein, allowing positive identification of the strain as *Haemophilus influenza*, and the phase status of the methylases HsdM and ModA, as well as the *modA* allele present in each of the paired isolates is included. This latter criteria was also used to identify paired isolates as the same strain. Numbers denote the number of repeats followed by the percentage ON/OFF. For example, using the *lgtC* gene in strain 217, 43 @ 82% = 43 GACA repeats at 82%; therefore gene is ON. If no percentage is shown, the gene is >95% ON or OFF. The colour indicates whether the gene is majority ON (green), OFF (RED) or mixed (orange). Blue indicates the gene is not phasevariable (‘0’ repeats). Blank cells indicate no data was collected.
(PDF)

Table S1 **NTHi strains used in this study.** ^*^strains recovered during the period 1982–1986; ^#^strains recovered during the period 2004–2008.
(DOCX)

Table S2 Primers used in this study to amplify repeat regions in phase variable LOS genes.
(DOCX)

Table S3 **Primers used in this study to amplify other genes.** OMP P6 gene, DNA Recognition Domain (DRD) of *modA* gene, OMP P2 gene, and OMP P5 gene.
(DOCX)
