# Supplementary material for: Selection for Phase Variation of LOS Biosynthetic Genes Frequently Occurs in Progression of Non-Typeable Haemophilus influenzae Infection from the Nasopharynx to the Middle Ear of Human Patients
Source: PLoS One. 2014 Feb 28;9(2):e90505. doi: 10.1371/journal.pone.0090505 (PMC3938747; doi:10.1371/journal.pone.0090505)
Supplement: Figure S1 — All data generated by fragment analysis of the seven phasevariable LOS synthesis genes, summarized in Figure 2 . In addition to the raw data presented as the heat map in Figure 2, the status of the P6 protein, allowing positive identification of the strain as Haemophilus influenza, and the phase status of the methylases HsdM and ModA, as well as the modA allele present in each of the paired isolates is included. This latter criteria was also used to identify paired isolates as the same strain. Numbers denote the number of repeats followed by the percentage ON/OFF. For example, using the lgtC gene in strain 217, 43 @ 82% = 43 GACA repeats at 82%; therefore gene is ON. If no percentage is shown, the gene is >95% ON or OFF. The colour indicates whether the gene is majority ON (green), OFF (RED) or mixed (orange). Blue indicates the gene is not phasevariable (‘0’ repeats). Blank cells indicate no data was collected. (PDF) [file pone.0090505.s001.pdf]

Isolate	Site of isolation	lgtC	lex2A	lic2A	lic1A	lic3A	lic3B	oafA	mod group	mod	hsdM	Hi P6
214	NP	no gene	15	18 @ 89%	24 @ 92%	22 @ 93%	no gene	0	modA4	18	8	+
1370	MEE	no gene	15	18 @ 94%	24 @ 92%	22 @ 92%	no gene	0	modA4	17 @ 85%	8	+
1371	MEE	no gene	15	21 @ 94%	24 @ 85%	22 @ 93%	no gene	0	modA4	16 @ 82%	8 @ 87%	+
217	NP	43 @ 82%	31 @ 84%	31	42 @ 84%	44	no gene	0	modA2	1	2	+
1380	MEE	43 @ 85%	31 @ 84%	31	42 @ 86%	44	no gene	0	modA2	1	2	+
284	NP	30 @ 89%	no gene	16	38	36	no gene	20 @ 88%	modA10	10 @ 83%	10 @ 89%	+
1657	MEE	31 ON @ 55%	no gene	16	38	36	no gene	20 @ 70%	modA10	11	10 @ 92%	+
287	NP	30	26 @ 87%	16	22	23	12	14	modA4	17 @ 90%	2	+
1666	MEE	30	25 @ 84%	16	22	23	12	14	modA4	17 @ 90%	2	+
1667	MEE	30	26 @ 86%	16	22 @ 94%	24 @ 92%	12	14	modA4	18	2	+
183	NP	17	no gene	14	34 @ 89%	25 @ 90%	16	14	modA2	3	5	+
1236	MEE	17	no gene	13	34 @ 89%	25 @ 91%	16	14 @ 94%	modA2	3	5	+
166	NP	27	19 OFF @ 34%	27	34 @ 86%	32	20 @ 92%	11	modA5	3	4 @ 94%	+
1182	MEE	27	20 @ 89%	27	34 @ 89%	32	20 @ 94%	12	modA5	3	5 @ 94%	+
172	NP	no gene	25 @ 75%	22	42 @ 70%	14	11	8	modA7	3	1	+
1199	MEE	no gene	26	22	42 @ 72%	13	10	9	modA7	3	1	+
180	NP	no gene	25 @ 87%	16	49@ 90%	21 @ 94%	no gene	11	modA11	32	3 @ 87%	+
1230	MEE	no gene	26 @ 88%	16	50 (&49)	22 @ 93%	no gene	10	modA11	8	5	+
182	NP	28 @ 90%	17 @ 91%	7	12 @ 93%	23	39 @ 85%	3	modA10	7	3 @ 92%	+
1234	MEE	29 @ 90%	17	7	12	21 @ 91%	38 @ 88%	3	modA10	7	3 ON @ 38%	+
297	NP	16	13	17	15 @ 92%	31 @ 91%	15 @ 94%	14	modA3	3	5	+
1714	MEE	16	13 @ 76%	17 @ 92%	15 @ 94%	31 @ 89%	16	14 @ 89%	modA3	3	3 @ 75%	+
1715	MEE	16	13	17 @ 93%	15	31 @ 90%	15 @ 93%	14 @ 94%	modA3	3	5 @ 74%	+
1848NP	NP	21	24	13	27 @ 78%	28 @ 73%	no gene	0	modA7	3	1	+
1848L	MEE	22	24	13 @ 84%	25 @ 87%	17 @ 70%	no gene	0	modA7	3	1	+
1885NP	NP	13	33	8	30 @ 89%	25 @ 92%	9	11 @ 88%	modA6	3	5	+
1885R	MEE	13	33	14	30 @ 84%	28 @ 89%	10 @ 87%	11	modA6	3	5	+
6N	NP	31 @ 93%	9	19	43 @ 84%	31	no gene	0	modA5	10	5	+
6E	MEE	31 @ 93%	9	19	43 @ 82%	32	no gene	0	modA5	10	5	+
20N	NP	41 @ 80%	24	10	57 ON @ 51%	14	no gene	0	modA10	23 @ 90%	6 @ 85%	+
20E	MEE	40 ON @ 44%	25	10	57 ON @ 37%	14	no gene	0	modA10	23 @ 90%	4&5 @ 93%	+
35N	NP	37 @ 90%	16	22	18 @ 88%	37	22	17 @ 91%	modA7	35	3 @ 85%	+
35E	MEE	36	16	22	18	37	22	17 @ 80%	modA7	35	2 @ 80%	+
48N	NP	9	16	13	27 @ 79%	18	15	9	modA6	3	2	+
48E	MEE	9	16	13	27 @ 86%	18	15	9	modA6	3	2	+
69N	NP	32 @ 85%	46 @ 75%	33 @ 87%	21 ON @ 43%	33	no gene	0	modA8	14&15	6 @ 84%	+
69E	MEE	32 @ 84%	46 @ 77%	35 @ 79%	21 ON @ 43%	33	no gene	0	modA8	13 ON @ 66%	8 @ 82%	+
70N	NP	32 @ 85%	12	17 @ 94%	34 @ 87%	28	no gene	20 @ 86%	modA4	1	8	+
70E	MEE	32 @ 84%	12	17 @ 96%	34 @ 90%	28	no gene	20 @ 87%	modA4	1	8 @ 90%	+
73N	NP	21	19 @ 87%	19	21 @ 83%	18	25	11	modA5	3	5 @ 91%	+
73E	MEE	21	17 @ 93%	19	21 @ 86%	18	23	10	modA5	3	4	+
111N	NP	27	3	19	24 @ 86%	23	11	8	modA2	9	2	+
111E	MEE	27	3	19	24 @ 82%	23	11	8	modA2	9	2	+
140N	NP	34 @ 75%	23 @ 88%	16	43 @ 78%	21	13	0	modA2	9 @ 89%	2	+
140E	MEE	35 @ 81%	23 @ 90%	16	42 @ 71%	21	13	0	modA2	9	2	+
138N	NP								modA6			

188E	MEE								modA8			
266	NP								modA3			
1590	MEE								modA5			
10567&8NP	NP								modA2			
10567R	MEE								modA7			
10568L	MEE								modA7			
86-027NP	NP								modA2			
86-027L	MEE								modA3			
86-027R	MEE								modA3			
50N	NP								no data			
50E	MEE								nodata			
109N	NP								no data			
109E	MEE								nodata			
147N	NP								no data			
147E	MEE								nodata			
74N	NP	18	23	26	27			17	modA2	13	2	
74E	MEE	21	no gene	20	20			10	modA2	3	4	
86-017NP	NP	no gene	21	19	30 @ 88%	20	12	10	modA7	3	1	+
86-017R	MEE	no gene	20 @ 92%	20 @ 94%	30	20	12	10	modA7	3	1	+
