# Supplementary material for: Selection for Phase Variation of LOS Biosynthetic Genes Frequently Occurs in Progression of Non-Typeable Haemophilus influenzae Infection from the Nasopharynx to the Middle Ear of Human Patients
Source: PLoS One. 2014 Feb 28;9(2):e90505. doi: 10.1371/journal.pone.0090505 (PMC3938747; doi:10.1371/journal.pone.0090505)
Supplement: Table S1 — NTHi strains used in this study. *strains recovered during the period 1982–1986; #strains recovered during the period 2004–2008. (DOCX) [file pone.0090505.s002.docx]

**Table S1.** NTHi strains used in this study.

| **Strain** |  | **Site of isolation (NP nasopharyngeal isolate; MEE middle ear effusion)** | ***modA* allele** | **Same OMP P2 gene?** | | **Same OMPP5 gene?** | | **Same**  **strain and thus further study?** |
| --- | --- | --- | --- | --- | --- | --- | --- | --- |
| 214 | * | NP |  | yes | | yes | | yes |
| 1370 |  | MEE | modA4 |  |  |  |  |  |
| 1371 |  | MEE |  |  |  |  |  |  |
| 217  1380 | * | NP  MEE | modA2 | yes | | yes | | yes |
| 284  1657 | * | NP  MEE | modA10 | yes | | yes | | yes |
| 287  1666  1667 | * | NP  MEE  MEE | modA4 | yes | | yes | |  |
|  |  |  |  |  |  |  |  | yes |
|  |  |  |  |  |  |  |  |  |
| 183 | * | NP | modA2 | yes | | yes | | yes |
| 1236 |  | MEE |  |  |  |  |  |  |
| 166 | * | NP | modA5 | yes | | yes | | yes |
| 1182 |  | MEE |  |  |  |  |  |  |
| 172 | * | NP | modA7 | yes | | yes | | yes |
| 1199 |  | MEE |  |  |  |  |  |  |
| 180 | * | NP | modA11 | yes | | yes | | yes |
| 1230 |  | MEE |  |  |  |  |  |  |
| 182 | * | NP | modA10 | yes | | yes | | yes |
| 1234 |  | MEE |  |  |  |  |  |  |
| 297 | * | NP | modA3 | yes | | yes | | yes |
| 1714 |  | MEE |  |  |  |  |  |  |
| 1715 |  | MEE |  |  |  |  |  |  |
| 1848NP | * | NP | modA7 | yes | | yes | | yes |
| 1848L |  | MEE |  |  |  |  |  |  |
| 1885NP | * | NP | modA6 | yes | | yes | | yes |
| 1885R |  | MEE |  |  |  |  |  |  |
| 6N | # | NP | modA5 | yes | | yes | | yes |
| 6E |  | MEE |  |  |  |  |  |  |
| 20N | # | NP | modA10 | yes | | yes | | yes |
| 20E |  | MEE |  |  |  |  |  |  |
| 35N | # | NP | modA7 | yes | | yes | | yes |
| 35E |  | MEE |  |  |  |  |  |  |
| 48N | # | NP | modA6 | yes | | yes | | yes |
| 48E |  | MEE |  |  |  |  |  |  |
| 69N | # | NP | modA8 | yes | | yes | | yes |
| 69E |  | MEE |  |  |  |  |  |  |
| 70N | # | NP | modA4 | yes | | yes | | yes |
| 70E |  | MEE |  |  |  |  |  |  |
| 73N | # | NP | modA5 | yes | | yes | | yes |
| 73E |  | MEE |  |  |  |  |  |  |
| 111N | # | NP | modA2 | yes | | yes | | yes |
| 111E |  | MEE |  |  |  |  |  |  |
| 140N | # | NP | modA2 | yes | | yes | | yes |
| 140E |  | MEE |  |  |  |  |  |  |
| 138N | # | NP | modA6 | NO DATA | | NO DATA | | no |
| 138E |  | MEE | modA8 |  |  |  |  |  |
| 266  1590 | * | NP  MEE | modA3 | NO DATA | | NO DATA | | no |
|  |  |  | modA5 |  |  |  |  |  |
| 10567&8NP  10567R  10568L | * | NP  MEE  MEE | modA2 | NO DATA | NO DATA | | no | |
|  |  |  | modA7 |  |  |  |  |  |
|  |  |  | modA7 |  |  |  |  |  |
| 86-027NP  86-027L  86-027R | * | NP  MEE  MEE | modA2 | NO DATA | NO DATA | | no | |
|  |  |  | modA3 |  |  |  |  |  |
|  |  |  | modA3 |  |  |  |  |  |
| 50N  50E | # | NP  MEE | NO DATA | NO DATA | NO DATA | | no | |
| 109N  109E | # | NP  MEE | NO DATA | NO DATA | NO DATA | | no | |
| 147N  147E | # | NP  MEE | NO DATA | NO DATA | NO DATA | | no | |
| 74N  74E | # | NP  MEE | modA2 | NO DATA | NO DATA | | no – highly variable | |
| 86-017NP  86-017R | * | NP  MEE | modA7 | no | yes | | no – unmatched OMP P2 gene | |
