# Supplementary material for: Selection for Phase Variation of LOS Biosynthetic Genes Frequently Occurs in Progression of Non-Typeable Haemophilus influenzae Infection from the Nasopharynx to the Middle Ear of Human Patients
Source: PLoS One. 2014 Feb 28;9(2):e90505. doi: 10.1371/journal.pone.0090505 (PMC3938747; doi:10.1371/journal.pone.0090505)
Supplement: Table S2 — Primers used in this study to amplify repeat regions in phase variable LOS genes. (DOCX) [file pone.0090505.s003.docx]

**Table S2 –** Primers used in this study to amplify repeat regions in phase variable genes

| **Gene** | **Repeat unit** | **Reps ON or OFF example** | **Primers** | **refs** |
| --- | --- | --- | --- | --- |
| *lgtC* | GACA | 10 = ON;  11 & 12 = OFF | For: 5’-VIC-TCATCGAGCAAAGGCATTG-3’  Rev: 5’-CTTACAGCTAAATAAGGTGC -3’^a^  Rev (2) 5’- TACTTACAGCTAAATAAG-3’^a^ | This study |
| *lex2A* | GCAA | 10 & 11 = OFF; 12 = ON | For: 5’-NED-CGGAATTATGTTAATCAC-3’  Rev: 5’-GTTTGCTTTGTGATGTAC-3’ | This study |
| *lic2A* | CAAT | 10 = ON;  11 & 12 = OFF | For: 5’-FAM-ACTGAACGTCGCAAA-3’  Rev: 5’-GCTAATTAAACAGCCT-3’ | High *et al*, 1993 |
| *lic1A* | CAAT | 10 & 11 = OFF; 12 = ON | For: 5’-VIC-CAAAAATAACTTTAACGTG-3’  Rev: 5’-AATGCTGATGAAGAAAATG-3’ | This study |
| *lic3A* | CAAT | 10 & 11 = OFF; 12 = ON | For: 5’-NED-ATTACCTGCAATAATGACAG-3’  Rev: 5’-TATTCAATGAACGGTAGAAT-3’  Lic3A specific: 5’GCCAGTAGTCGCAAAAGTGTC-3’ | Van Belkum *et al*, 1997; this study |
| *lic3B* | CAAT | 11 = ON;  12 & 13 = OFF | For: 5’-NED-ATTACCTGCAATAATGACAG-3’  Rev: 5’-TATTCAATGAACGGTAGAAT-3’  Lic3B specific: 5’TCAAACATCTTGCCGTCTTC-3’ | Van Belkum *et al*, 1997; this study |
| *oafA* | GCAA | 9 & 10 = OFF; 11 = ON | For: 5’-FAM-GCCTAATATTTATTATCTCTC-3’  Rev: 5’-GTATGAATAATTAATGCTG-3’ | Fox *et al*, 2005 |
| *modA* | AGCC or AGTC | 10 = ON;  11 & 12 = OFF | For: 5’-FAM- ATGGCGGGCAAAGCACCGAAGA-3’  Rev: 5’- CAAAAAGCCGGTCAATTTCATCAAA-3’ | Srikhanta *et al*, 2005 |
| *hsdM* | GAGAC | 3 = ON;  4 & 5 = OFF | For: 5’-VIC- ACGCAAAAATCCCTTTCAACC-3’  Rev: 5’-GACCAAGAACGATATGTTTATAG-3’ | This study |

^a^ different downstream regions in different strains; primers give same sized product with forward primer
