# Supplementary material for: Selection for Phase Variation of LOS Biosynthetic Genes Frequently Occurs in Progression of Non-Typeable Haemophilus influenzae Infection from the Nasopharynx to the Middle Ear of Human Patients
Source: PLoS One. 2014 Feb 28;9(2):e90505. doi: 10.1371/journal.pone.0090505 (PMC3938747; doi:10.1371/journal.pone.0090505)
Supplement: Table S3 — Primers used in this study to amplify other genes. OMP P6 gene, DNA Recognition Domain (DRD) of modA gene, OMP P2 gene, and OMP P5 gene. (DOCX) [file pone.0090505.s004.docx]

**Table S3 –** Primers used in this study to amplify *ompP6* gene, DNA Recognition Domain (DRD) of *modA* gene, and the *ompP2* and *ompP5* genes

| **Gene** | **Primer name** | **Primer sequence** | **Reference** |
| --- | --- | --- | --- |
| *ompP6* | P6-F  P6-R | 5’ATGAACAAATTTGTTAAATCA  5’TTAGTACGCTAACACTGC | Murphy *et al*., 2007 |
| *mod* | him6a  him11 | 5’GCAGTTTAAAGGTAAAGTTAAGC  5’ TCGTTAAATGGAGCAAGTTC | Fox *et al*., 2007 |
| *ompP2* | P2-F  P2-R | 5’ CACTTGCAGCATTAATCGTTGGTGC  5’ GTAAACGCGTAAACCTACACCCAC | This work |
| *ompP5* | P5-F  P5-R | 5’ CTGCAATCGCATTAGTAGTTGCTGG  5’ GTACCGTTTACCGCGATCTCTAC | This work |
